# United States Consul-General J. H. Worman

**Published:** 1903-02

**Authors:** 


### UNITED STATES CONSUL-GENERAL J. H. WORMAN.

UNDER the heading of Foreign Correspondence will be found a letter from Consul Worman to the editor, and also a copy of a letter sent to Dr. Crouse, of Chicago. In these two letters Consul Worman complains that justice has not been given him, and further that false charges have been made involving his integrity. He very properly seeks redress, and demands that the question of the disposition of the moneys collected by the Association of Dental Faculties and the National Dental Association should be settled to his satisfaction and that of the dental profession.

The writer has never been able to understand the statement made that Consul Worman's evidence was not of much value. From a legal stand-point this may have been true, but the manner in which this evidence was treated seems to justify the opinion that further explanation is needed.

Consul Worman never received anything to remunerate him for his services in collecting evidence abroad regarding the diploma traffic, and whether this was valuable or otherwise, it was largely through his personal efforts that the condition of things in Illinois and the State board was brought to the attention of the two societies named, and the money raised. The committee having this fund in charge should, at least, comply with Consul Worman's wishes and relieve him of the unjust charge that all his work was "bought testimony."

